# Epidemiology and genotyping of *Anaplasma marginale* and co-infection with piroplasms and other *Anaplasmataceae* in cattle and buffaloes from Egypt

**DOI:** 10.1186/s13071-020-04372-z

**Published:** 2020-09-29

**Authors:** Amira AL-Hosary, Cristian Răileanu, Oliver Tauchmann, Susanne Fischer, Ard M. Nijhof, Cornelia Silaghi

**Affiliations:** 1 Department of Animal Medicine (Infectious Diseases), Faculty of Veterinary Medicine, Assiut University, Assiut, 71526 Egypt; 2 Institute of Infectology, Friedrich-Loeffler-Institut, Federal Research Institute for Animal Health, Sudufer 10, 17493 Greifswald-Insel Riems, Germany; 3 Freie Universität Berlin, Institute of Parasitology and Tropical Veterinary Medicine, Berlin, 14163 Germany; 4 Department of Biology, University of Greifswald, Domstrasse 11, 17489 Greifswald, Germany

**Keywords:** *Anaplasma marginale*, Cattle, Buffaloes, Diagnostic tools, Genotypes, Co-infections, Egypt

## Abstract

**Background:**

*Anaplasma marginale* is an obligate intracellular bacterium and the main cause of bovine anaplasmosis in tropical and subtropical regions. In Egypt, data regarding the prevalence of *A. marginale* in ruminant hosts and of the circulating genotypes is lacking. This study therefore aimed to (i) investigate the presence, epidemiology and genotypes of *A. marginale* in cattle and buffaloes in Egypt, (ii) to evaluate suitable diagnostic tools and (iii) to identify co-infections of *A. marginale* with other selected tick-borne pathogens.

**Methods:**

Blood samples were collected from 394 animals (309 cattle and 85 buffaloes) from three different areas in Egypt. For the detection of *A. marginale* infection, several tests were compared for their sensitivity and specificity: blood smear analysis, enzyme-linked immunosorbent assay (ELISA), PCR, real-time PCR and reverse line blot (RLB) assay. Co-infections with *A. marginale,* piroplasms and other *Anaplasmataceae* were surveyed by RLB while *A. marginale* genotypes were identified by amplifying and sequencing the partial *msp1α* gene.

**Results:**

*Anaplasma marginale* DNA was amplified by qPCR in 68.3% of cattle and 29.4% of buffaloes. RLB showed infection with *A. marginale* in 50.2% of cattle and 42.5% of buffaloes. Blood smear analysis detected this agent in 16.2% of cattle and 2.4% of buffaloes. ELISA showed specific antibodies against *A. marginale* in 54.9% of cattle. *Anaplasma marginale* was associated, in cattle and buffaloes, with several tick-borne pathogens (*Theileria annulata*, *Babesia bovis*, *Babesia bigemina*, *Babesia occultans* and *Anaplasma platys*). A significant difference of *A. marginale* infection level was noticed in cattle, where animals between 3–5-years-old had a higher prevalence (79.2%) compared to those older than 5 years (36.4%) and younger than 3 years (59.7%) and one year (64.5%), respectively (*P* = 0.002281). Microsatellite analysis identified 15 different genotypes.

**Conclusions:**

The epidemiological findings revealed high prevalence of *A. marginale* in cattle and buffaloes in all the investigated areas. The circulation of diverse genotypes was observed, most of these *A. marginale* genotypes being specific for Egypt. The qPCR assay was confirmed to be the most sensitive tool for detection of *A. marginale* in cattle and buffaloes even in the carrier state, highlighting the importance of using suitable diagnostic tests. 
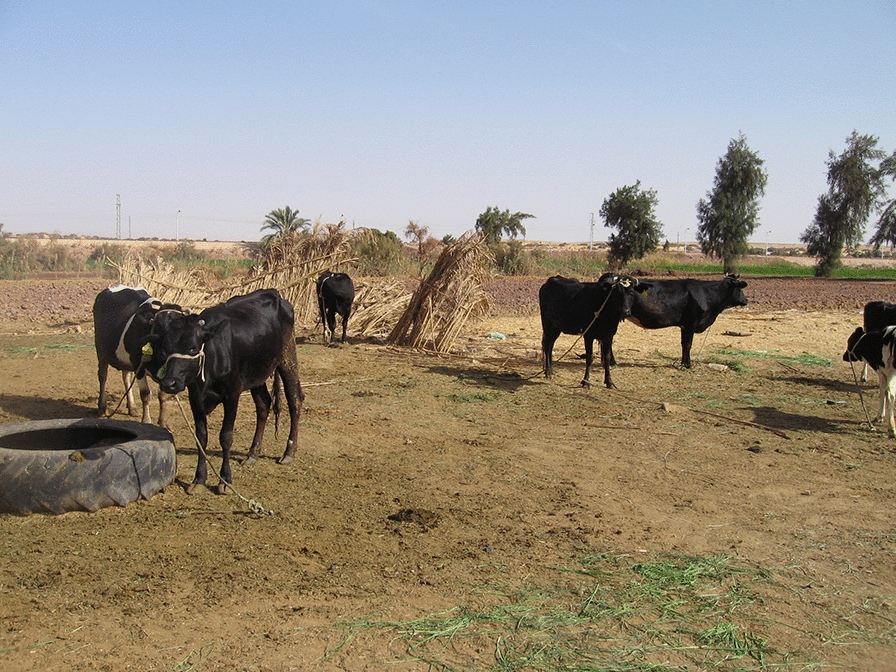

## Background

Tick-borne diseases (TBDs) are responsible for important health problems worldwide [1, 2]. In Egypt, TBDs cause major health disorders, in particular to exotic and cross-bred cattle, endangering the wellbeing of animals and the livelihood of their owners [3, 4]. Bovine theileriosis caused by *Theileria annulata* and bovine babesiosis caused by *Babesia bovis* and/or *Babesia bigemina* are the most common TBDs in Egypt [1, 5, 6]. They are among the main impediments of livestock production in Egypt due to the fact that both interfere with animal productivity [1, 5, 7, 8]. Bovine anaplasmosis is characterized by mild to severe hemolysis and anemia that adversely affects animal health, production and reproduction [9, 10]. It is caused by *Anaplasma marginale*, an intraerythrocytic rickettsia mainly transmitted by *Rhipicephalus microplus* ticks, but other tick species have also been incriminated as vectors worldwide [11, 12]. Mechanical transmission through contaminated needles or surgical instruments under poor hygienic conditions or through biting flies may also occur. Both tick and animal hosts are considered reservoirs for this pathogen and can become persistently infected with *A. marginale*. Co-infections with *A. marginale* and other tick-borne pathogens such as *Theileria*, *Babesia* and other *Anaplasma* species are common in cattle [4, 13–15].

The persistence of *A. marginale* infection is enabled by antigenic variation [9, 10, 16, 17]. The Major surface proteins (MSP) of *A. marginale* play an important role in the interaction with the host, as these are highly variable proteins and responsible for the invasion of host cells. This multigene protein family usually undergoes antigenic changes and the resulting amino acids (antigens) were found to be characteristic for each geographic area. In Egypt, data on the prevalence of *A. marginale* and the circulating genotypes are lacking. Furthermore, the tick species transmitting this pathogen are not fully characterized. However, we hypothesized that *A. marginale* is a major tick-borne pathogen in Egypt occurring frequently as a single infection or in co-infection with other pathogens in cattle and buffaloes.

This study therefore aimed to (i) investigate the presence, epidemiology and genotypes of *A. marginale* in cattle and buffaloes in Egypt, (ii) evaluate suitable diagnostic tools, and (iii) identify co-infections with *A. marginale* and other selected tick-borne pathogens.

## Methods

### Study areas

**Fig. 1:**
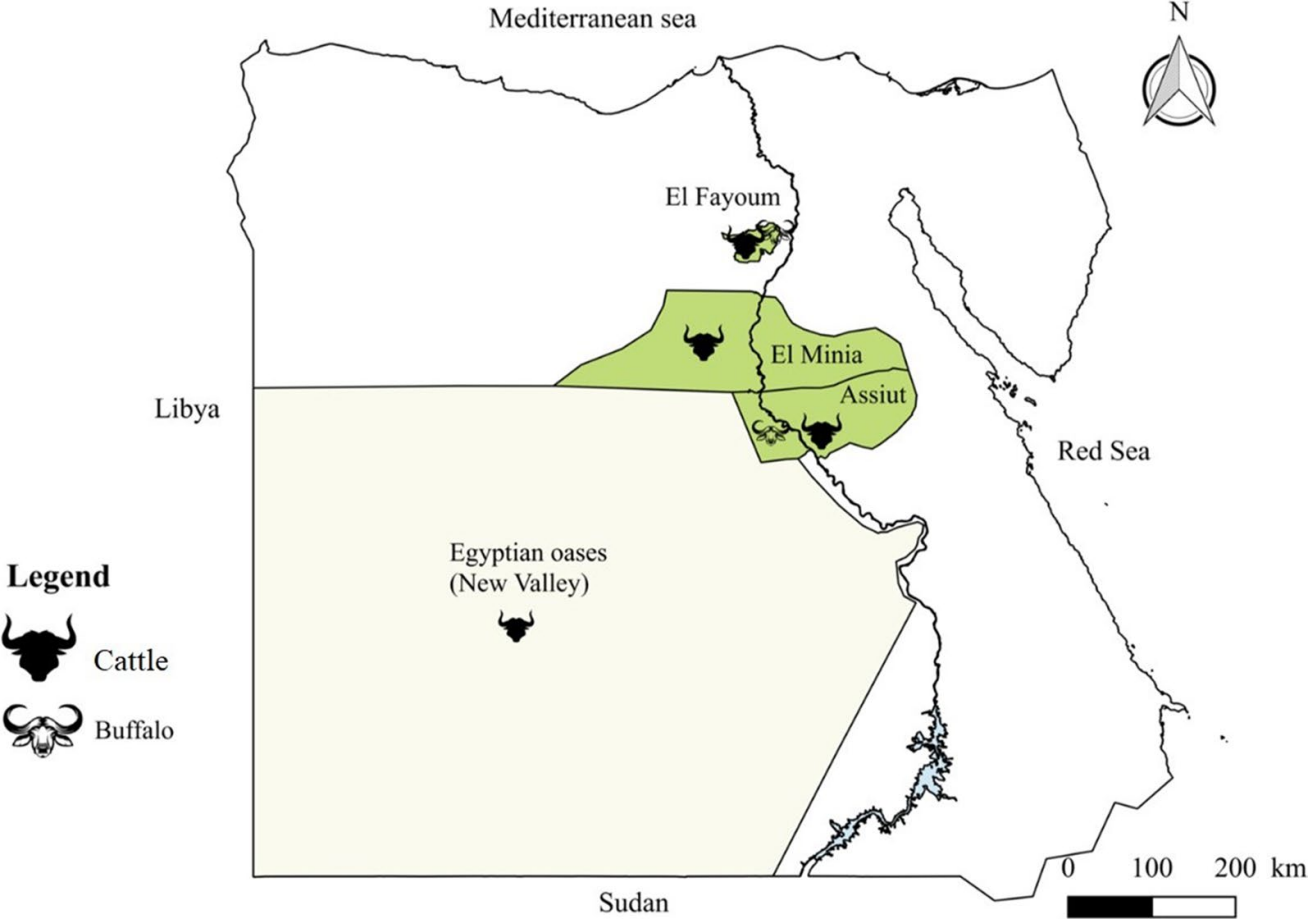
Map of Egypt indicating the study areas. Cattle samples were collected from three different regions in Egypt: Upper Egypt (EL-Minia and Assiut governorates); Middle Egypt (El-Fayoum); and Egyptian oases (New Valley). Collection of samples from buffaloes was performed in the Upper Egypt (only in Assiut Governorate) and in Middle Egypt (EL-Fayoum)

Samples were collected from cattle and buffaloes from three different regions in Egypt: Upper Egypt (EL-Minia and Assiut governorates), Middle Egypt (EL-Fayoum) and Egyptian oases (New Valley). Upper Egypt is a geographical and cultural division of Egypt running along the River Nile from Aswan northwards until the Delta. Middle Egypt is located between the Upper Egypt and Lower Egypt, from Assiut northwards until Memphis. The Egyptian oases is one of the biggest governorates in Egypt and represents more than 46% of the whole land area of Egypt. It is located at the plateau of the Egyptian western desert in southwestern Egypt and it borders Sudan to the south, River Nile to the east, and Libya to the west [18, 19] (Fig. 1).

### Sample collection

Samples were collected from January to December 2018. Clinical examination was carried out on all animals before sampling. The examination included the measurement of body temperature, pulse and respiratory rate [20]. Three sample categories were collected from each animal: one blood sample in EDTA tubes from the ear vein for preparation of blood smears, one EDTA blood sample from the jugular vein for DNA extraction and another blood sample in a plain tube for serum preparation [21]. Inspection of the animal’s coats, udder, scrotum, inner side of the thighs and dewlap was performed for presence of ticks [20]. Tick samples were collected in 15 ml dry-screw cap Falcon™ tubes then transferred to 2 ml tubes containing 70% ethanol for preservation [22].

### Tick identification

Ticks were identified to the species level using morphological identification keys under a ZEISS Stemi 508 stereomicroscope (Carl Zeiss, Oberkochen, Germany) [22, 23].

### Microscopical examination

Thin blood smears were prepared and stained by Giemsa stain (Sigma-Aldrich, G4507, Darmstadt, Germany), then examined for the presence of blood pathogens under a light microscope (Olympus BX3M, Tokyo, Japan) using oil-immersion lens at a magnification of 1000× [21].

### Serological diagnosis

Antibody detection was carried out by enzyme-linked immunosorbent assay (ELISA) on serum samples from cattle for detection of specific antibodies against *A. marginale* by using a commercial kit (SVANOVIR^®^ *A. marginale*, Svanova, Uppsala, Sweden), according to the manufacturer’s instructions.

### Molecular detection

#### DNA extraction

DNA extraction from blood of cattle and buffaloes was performed using the QIAamp DNA Blood kit (Qiagen, Hilden, Germany) according to the manufacturerʼs instructions.

#### Molecular detection of Anaplasma marginale by real time PCR

*Anaplasma marginale* was detected in cattle and buffalo samples by real time PCR (qPCR) targeting the *msp1β* gene [24]. The reaction was done in a total volume of 25 µl which included 12.5 µl (2×) iTaq^TM^ Supermix (Bio-Rad Laboratories, Feldkirchen, Germany), 0.9 µl of molecular grade water, 0.5 µl of each primer (10 µM), 0.6 µl of the probe (5 µM) and 10 µl of DNA template. The qPCR program included an initial denaturation step at 95 ℃ for 10 min followed by 45 cycles of amplification at 95 ℃ for 45 s and 60 ℃ for 60 s then cooling. A positive control (*A. marginale* DNA from cattle, kindly provided by Dr Sándor Hornok), and a negative control (water) were added with each reaction.

### Reverse line blot hybridization assay

Reverse line blot hybridization assay (RLB) was performed as previously described [13] for the simultaneous detection of several tick-borne pathogens including *Theileria* spp., *Babesia* spp., *Anaplasma* spp., *Ehrlichia* spp., *Rickettsia* spp. and *Midichloria mitochondrii*.

### Conventional PCRs and sequencing

#### Anaplasmataceae

For confirmation of the RLB results, additional PCRs were performed to identify the *Anaplasma* species detected in some of the samples. A conventional PCR using GoTaq® Flexi DNA Polymerase kit (Promega, Madison, USA) was done to amplify a 855-bp fragment of the *groEL* gene of *Anaplasma* spp.

Since the GroEL PCR did not yield positive results in buffalo samples, a semi-nested PCR targeting the *16S* rRNA gene of *Anaplasma/Ehrlichia* spp. was performed for the amplification of a fragment of 426 bp as described elsewhere [25] (Table 1). The PCR products were run on a 1.5% agarose gel stained with Roti®-Gel Stain Red (Carl Roth GmbH, Karlsruhe, Germany) for 40 min at 75 V and visualized with ChemiDoc^TM^ MP Imaging system (Bio-Rad Laboratories, Hercules, USA).

**Table 1:** Primers used for confirmation of co-infections and identification of *Anaplasma marginale* genotypes

Pathogen	Amplified gene	Primer sequences (5’–3’)	Fragment size (bp)	Annealing T (℃)	References
*Anaplasma* spp.	*GroEL*	GroEL‐F2: ATG(GT)CAAATACGGT(AT)GTCACGG	855	62	This study
		GroEL‐R8: TCRCCAAGCATRTCYTTTCTTC			
*Anaplasma/ Ehrlichia* spp.	*16S* rRNA	FD1: AGAGTTTTGATCCTGGCTCAG*	426	55	[25]
		EHR1: TAGCACTCATCGTTTACAGC			
		GA1UR: GAGTTTGCCGGGACTTCTTCT			
*Babesia* spp.	*18S* rRNA	BJ1: GTCTTGTAATTGGAATGATGG	400–500	55	[26]
		BN2: TAGTTTATGGTTAGGACTACG			
*Theileria* spp.	*18S* rRNA	THfor: TGACACAGGGAGGTAGTGA	500	65	[27]
		THrev: TCAGCCTTGCGACCATACT			
*A. marginale*	*MSP1α* 1st PCR *MSP1α* 2nd PCR	1733F: TGTGCTTATGGCAGACATTTCC 3134R1: TCACGGTCAAAACCTTTGCTTACC	800–1000	55	[9]
		1733F: TGTGCTTATGGCAGACATTTCC 2957R2: AAACCTTGTAGCCCCAACTTATCC		60	

*Abbreviation*: T, temperature

#### *Babesia*/*Theileria* species

*Babesia* and *Theileria* species were confirmed using two different primer pairs for each agent, targeting the *18S* rRNA gene. *Babesia* spp. was detected using the previously described method by Casati et al. [26]. For detection of *Theileria* species, a *Theileria-*specific primer pair was used [27] (Table 1). The composition of the PCR mix and gel electrophoresis for both *Babesia* and *Theileria* were identical as described above for *Anaplasma GroEL* PCR.

#### Genotyping of *Anaplasma marginale*

A semi-nested PCR targeting the *msp1α* gene was carried out on 19 DNA samples from cattle and buffaloes that tested positive for *A. marginale* by qPCR, registering CT values ≤ 25 cycles [11, 16] (Table 1). The first PCR was done on both cattle and buffalo samples. Clear strong specific bands (size ranging from 800 to 1000 bp) were obtained from cattle samples while buffalo samples were subjected to the second amplification. The PCR reactions were performed using GoTaq® Flexi DNA Polymerase kit (Promega, Madison, USA) as previously described [11, 16, 17, 25, 28].

### Sequencing

PCR sequencing reactions were performed for both forward and reverse strands. Reaction mix with a total volume of 10 µl included 1 µl 5× sequence buffer, 2 µl Big Dye ready for use master mix (Thermo Fischer, Darmstedt, Germany), 1 µl of each 10 µM primer and 5 µl molecular grade water. The thermal profile was 96 ℃ for 1 min as a primary denaturation, followed by 25 cycles of 96 ℃ for 10 s for denaturation, annealing temperature for 5 s depending on each used primer, 60 ℃ for 60 s for extension, and a final extension step at 72 ℃ for 5 min [29]. The PCR products were purified with NucleoSEQ® kit (Mackerey Nigel, Düren, Germany) according to the manufacturer’s instructions. After purification, 15 µl of each sample were mixed with 15 µl of the highly deionized (Hi-Di) formamide in 1.5 ml Eppendorf tube and sequenced on ABI PRISM® 3130 sequencer at the Institute of Diagnostic Virology, Friedrich-Loeffler-Institut, Germany.

### Phylogenetic and microsatellite analysis of *Anaplasma marginale msp1a* gene

The obtained sequences were analyzed with Geneious 11.1.5 (https://www.geneious.com). The similarity search of the truncated sequences was carried out by using BLAST analysis (http://blast.ncbi.nlm.nih.gov/) after removal of the primer sequences. Nucleotide sequences of each sample were translated into amino acid sequences by the Open Reading Frame Finder translation tool on NCBI (https://www.ncbi.nlm.nih.gov/orffinder/). Nucleotide and protein sequences were aligned using the multiple-alignment program ClustalW [30]. The phylogenetic analysis was inferred using the Neighbor-Joining method [31]. The evolutionary distances were computed using the Kimura 2-parameter model [32]. Evolutionary analyses were conducted in MEGA X using *A. phagocytophilum* (HG528610) as the outgroup [33].

The obtained Egyptian *A. marginale* sequences were classified depending on the microsatellite (G/A TTT) m (GT) n in the 5’UTR region, located between the putative Shine-Dalgarno (SD) sequence (GTAGG) and the translation initiation codon (ATG). The SD-ATG distance was calculated in nucleotides as (4 × m) + (2 × n) + 1 [28, 34, 35].

### Statistical analysis

Data were compared with Chi-square test using R in R Studio [36, 37]. Parameters related to animals such as age, breed and sex were investigated to find out the risk factors that may affect the animal susceptibility. In addition, some environmental factors like seasonal variation (hot months from April to September and non-hot months from October to March) and geographical areas (Upper Egypt, Middle Egypt and Egyptian oases) were taken in consideration during this study. The differences were considered significant at 5% threshold values. Detection tools for identification of *A. marginale* including blood smear, ELISA and RLB were evaluated against qPCR as a gold standard assay to calculate sensitivity, specificity, positive predictive value (PPV), negative predictive value (NPV) and combined predictive value (CPV) of each assay in both cattle and buffaloes [38].

## Results

A total number of 309 cattle (140 males and 169 females) and 85 buffaloes (35 males and 50 females) were sampled during this study. Animals originated from three different localities: Middle Egypt (119 cattle and 23 buffaloes), Upper Egypt (111 cattle and 62 buffaloes) and Egyptian Oases (79 cattle). All examined animals were apparently healthy and were infested with adults (male and female) and nymphs of two tick species, *Hyalomma excavatum* and *Rhipicephalus annulatus*.

**Fig. 2:**
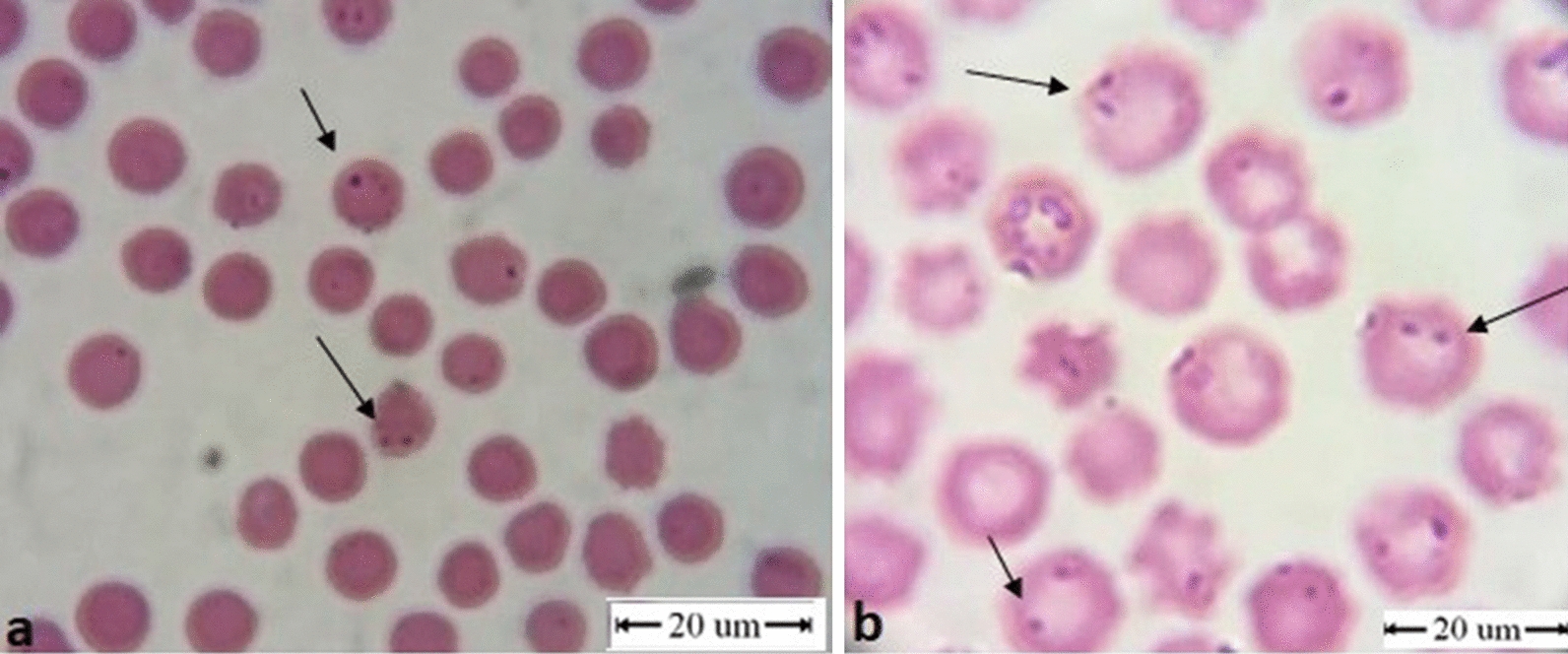
Blood smear showing *A. marginale* inside the red blood cells (arrows) (**a**) and *A. marginale *+* Babesia* spp. co-infection inside the red blood cells (arrows) showing anisocytosis and macrocytic hypochromic anaemia as a result (**b**)

*Anaplasma marginale* was observed in 16.2% (50/309) and 2.4% (2/85) of the blood smears from cattle and buffalo, respectively (Fig. 2). Antibodies against *A. marginale* were detected in 54.8% (103/188) of the cattle serum samples. Infection rates of 68.3% (211/309) in cattle and 29.4% (25/85) in buffalo were found by qPCR. RLB showed infection with *A. marginale* in 50.2% (155/309) of cattle samples and 42.4% (36/85) of buffalo samples (Table 2). The RLB showed co-infections with *A. marginale* and other *Anaplasma* species and with piroplasms in both cattle and buffaloes. This was confirmed by sequencing of the RLB-PCR products. Co-infection with *T. annulata* was recorded in 49 (15.9%) cattle. Co-infection with *B. bovis*, *B. bigemina* and *B. occultans* was detected in 18 (5.8%), 2 (0.7%) and 1 (0.3%) cattle, respectively. Co-infection of *A. marginale* + *A. platys* was detected in 26 (8.4%) of the cattle samples. Buffaloes tested positive for co-infections with *A. marginale* and *T. annulata* (*n* = 1; 1.18%), *B. bigemina* (*n* = 2; 2.35%) and *A. platys* (*n* = 4; 4.71%).

**Table 2:** Infection rates with *Anaplasma marginale* in cattle and buffaloes from Egypt

Species	Diagnostic tool	*n*/*N*	(%)
Cattle	Blood smear	50/309	16.2
	ELISA	103/188	54.8
	RLB	155/309	50.2
	qPCR	211/309	68.3
Buffaloes	Blood smear	2/85	2.4
	RLB	36/85	42.4
	qPCR	25/85	29.4

*Abbreviations*: ELISA, enzyme-linked immunosorbent assay; RLB, reverse line blot; qPCR, real time PCR; n, number positive; N, total number

The newly generated sequences from both cattle and buffalo were submitted to the GenBank database and are available under the following accession numbers: *T. annulata*: MN223723-MN223737; *B. bigemina*: MN227676-MN227679; *B. occultans*: MN227675; *A. platys*: MN202017-MN202023 and MN227688 and *A. marginale*: MN227687, MN227689-MN227692.

Real time PCR was considered as a gold standard detection assay in this study and according to the results obtained for *A. marginale* in both cattle and buffaloes, all detection assays were evaluated by assessing their sensitivity, specificity and predictive values (Table 3). The sensitivity of blood smear examination, ELISA and RLB assays in cattle was 23.7%, 66.7% and 60.7%, respectively, while in buffaloes the sensitivity of blood smears and RLB was 8.0% and 64.0%, respectively. On the other hand, the specificity of blood smear, ELISA and RLB in cattle was 100%, 78.0% and 84.7%, respectively, while in buffaloes the specificity of blood smear and RLB was 100% and 66.7%, respectively (Table 3).

**Table 3:** Evaluation of diagnostic assays for *Anaplasma marginale* against qPCR as a golden standard test

Species	Diagnostic tool	No. positive/No. total	No. negative/No. total	qPCR assay				Parameter				
				TP^d^	TN^e^	FP^f^	FN^g^	Sensitivity	Specificity	PPV^h^	NPV^i^	CPV^j^
Cattle	Blood smear	50/309	259/309	50	98	0	161	23.7	100	100	37.9	47.9
	ELISA^a^	103/188	85/188	92	39	11	46	66.7	78.0	89.3	45.9	69.7
	RLB^b^	155/309	154/309	128	83	15	83	60.7	84.7	89.5	50.0	68.3
Buffaloes	Blood smear	2/85	83/85	2	60	0	23	8.0	100	100	72.3	72.9
	RLB^b^	36/85	49/85	16	40	20	9	64.0	66.7	44.4	81.6	65.9

*Abbreviations*: ELISA, enzyme-linked immunosorbent assay; RLB, reverse line blot; qPCR, real time PCR; TP, true positive; TN, true negative; FP, false positive; FN, false negative; PPV, positive predictive value; NPV, negative predictive value; CPV, combined predictive value (CPV)

Sex, breed or locality did not influence the prevalence of *A. marginale*. In addition, seasonal variation (hot months and non-hot months) also did not influence *A. marginale* prevalence, but it varied with age of the animal. There was a statistically significant difference according to age in cattle but not in buffaloes (Tables 4, 5).

**Table 4:** Epidemiological parameters associated with *Anaplasma marginale* infection in cattle detected by *msp1β* qPCR (Chi-square test)

Parameter		*n*/*N*	%	*χ*^2^	*P*-value
Sex	Male	88/140	62.9	3.0389	0.08129
	Female	123/169	72.8		
Age	≤ 1 year	78/121	64.5	14.5160	0.00228**
	≤ 3 years	34/57	59.7		
	≤ 5 years	95/120	79.2		
	≥ 5 years	4/11	36.4		
Breed	Native	35/49	71.4	2.0257	0.3632
	Friesian	41/67	61.2		
	Crossbreed	135/193	69.9		
Season	Hot months	157/228	68.9	0.0508	0.8217
	Non-hot months	54/81	66.7		
Locality	Middle Egypt	83/119	69.8	0.3405	0.8435
	Upper Egypt	76/111	68.5		
	Egyptian Oases	52/79	65.8		

**P < 0.01

*Abbreviations*: n, number positive; N, total number

**Table 5:** Epidemiological parameters associated to *Anaplasma marginale* infection in buffaloes detected by *msp1β* qPCR (Chi-square test)

Parameter		*n*/*N*	%	*χ*^2^	*P*-value
Sex	Male	11/35	31.4	0.0099	0.9207
	Female	14/50	28.0		
Age	≤ 1 year	11/42	26.2	3.7649	0.2880
	≤ 3 years	7/14	50.0		
	≤ 5 years	2/6	33.3		
	≥ 5 years	5/23	21.7		
Season	Hot months	25/81	30.0	0.5783	0.4470
	Non-hot months	0/4	0		
Locality	Middle Egypt	6/23	26.1	0.0201	0.8872
	Upper Egypt	19/62	30.7		

*Abbreviations*: n, number positive; N, total number

*Anaplasma marginale* major surface protein α1 (*msp1α*) gene was sequenced for phylogenetic analysis and genotyping in both cattle and buffalo from different localities in Egypt. All sequences were also submitted to GenBank and can be retrieved using the following accession numbers: Middle Egypt (from cattle: MN273314, MN385279; from buffaloes: MN311245 and MN311246), Upper Egypt [Assiut governorate (from cattle: MN273312, MN273313 and MN385280; from buffaloes: MN 311243 and MN311244), EL-Minia governorate (MN257055: MN257057)] and Egyptian Oases (MN370071-MN370077).

The phylogenetic analysis revealed that the sequences for *A. marginale* group with different isolates from other countries or other local regions in Egypt (see Additional file 1: Figure S1).

*Msp1α* microsatellite sequences revealed that the isolates of *A. marginale* from Egypt are genetically different from other isolates reported worldwide and the microsatellite sequences produced SD-ATG distances between 23 and 27 nucleotides (Table 6). All the obtained sequences were translated into amino acids for assessing the tandems repeats (TR) in the Egyptian isolates. The results revealed that there are some amino acid repetitions specific for Egyptian *A. marginale* isolates. Twenty-seven TR sequences were found, 25 of which were identified for the first time in the Egyptian isolates and named as Eg1–Eg25. These TRs repeated between one to three times in each sample and resulted in the identification of 15 different genotypes in both cattle and buffaloes. The results also confirmed the occurrence of a predominant genotype in the collected samples with the following TRs sequence: Eg17, Eg23, Eg23, Eg23 and Eg24. This genotype was detected in cattle from two different areas including Upper Egypt (Assiut Governorate) and the Egyptian oases. On the other hand, two TRs (F) and (τ) matched with isolates from USA, Israel, Cuba and Brazil. The Egyptian isolate TR (F) is identical to the isolates from USA, Israel, Cuba and Brazil while the second isolate (τ) appeared with slight modification (Table 7).

**Table 6:** The msp1α microsatellite sequence analysis in *Anaplasma marginale* isolates from Egypt

Isolate	GenBank ID	Genotype	Structure of MSP1α tandem repeats	No. of repeats	m	N	SD-ATG distance
EL-Minia-43	MN257055	Minia-1	Eg21, Eg10, Eg11, Eg5	4	3	5	23
EL-Minia-44	MN257056	Minia-2	Eg10, Eg12, Eg13, Eg10	4	2	7	23
EL-Minia-45	MN257057	Minia-3	Eg14, Eg15, Eg16, Eg16, Eg16, Eg6	6	2	7	23
Assiut-59	MN273312	Assiut-1	Eg10, Eg11, Eg5	3	3	5	23
Assiut-105	MN385280	NV-1	Eg17, Eg23, Eg23, Eg23, Eg24	5	3	5	23
Assiut-214	MN273313	Assiut-2	Eg11, Eg11, Eg22, Eg22, Eg14	5	3	5	23
Assiut-Buffalo268	MN 311243	Assiut-3	Eg8, Eg1, Eg1, Eg2	4	3	5	23
Assiut-Buffalo269	MN311244	Assiut-4	Eg8, Eg3, Eg8, Eg4	4	3	5	23
EL-Fayoum-33	MN273314	Fayoum-1	Eg7, F, F, F	4	3	5	23
EL-Fayoum-185	MN385279	Fayoum-2	Eg3, Eg1, Eg3, Eg3	4	3	5	23
EL-Fayoum-Buffalo 288	MN 311245	Fayoum-3	Eg8, Eg5, Eg8, Eg20	4	3	5	23
EL-Fayoum-Buffalo 291	MN 311246	Fayoum-4	Eg8, Eg5, Eg8, Eg9	4	3	5	23
New Valley-136	MN370074	NV-1	Eg17, Eg23, Eg23, Eg23, Eg24	5	3	5	23
New Valley-311	MN370075	NV-1	Eg17, Eg23, Eg23, Eg23, Eg24	5	3	5	23
New Valley-133	MN370072	NV-1	Eg17, Eg23, Eg23, Eg23, Eg24	5	4	5	27
New Valley-134	MN370073	NV-1	Eg17, Eg23, Eg23, Eg23, Eg24	5	4	5	27
New Valley-2	MN370077	NV-2	τ, Eg19, Eg3, Eg25	4	3	7	27
New Valley-337	MN370071	NV-3	Eg17, Eg23, Eg18	3	3	7	27
New Valley-370	MN370076	NV-4	Eg23, Eg23, Eg23, Eg18	4	4	5	27

*Abbreviations*: m, the number of repetitions of the nucleotide sequence G/A TTT; n, the number of repetitions of the nucleotide sequence GT; SD-ATG distance, distance calculated in nucleotides as (4 × m) + (2 × n) + 1

**Table 7:** Amino acids repetition in the isolates of *Anaplasma marginale* from Egypt

Amino acids repetition	TRs name	Locality
TDSSSASGQQQESSVLSQSGQASTSSQLG	Eg 1	Present study
TDSSSASGQQQESSVLSQSGQASTSSQSG	Eg 2	Present study
TDSSSASGQQQESSVLSQSDQASTSSQLG	Eg 3	Present study
TDSSSASGQQQESGVLSQSGQASTSSQSG	Eg 4	Present study
TDSSSASGQQQESGVSSQSDQASTSSQLG	Eg5	Present study
TDSSSASGQQQESGVSSQSDASTSSQLG	Eg6	Present study
TDSSSASGQQQESSVSSQSGQASTSSQLG	F	USA, Israel & Cuba
TDSSSASGQQQESSVLSPSGQASTSSQLG	τ	Brazil
SGSSSASGQQQESSVLSQGGQASTSSQLG	Eg7	Present study
ADSSSASGQQQESSVLSQSGQASTSSQLG	Eg8	Present study
ADSSSASGQQQESGVLSQSGQASTSSQLG	Eg9	Present study
ADSSSASGQQQESGVSSQSDQASTSSQLG	Eg10	Present study
ADSSSASGQQQESGVPSQSGQASTSSQLG	Eg11	Present study
ADSSSASGQQQESGVSSQSSHASTSSQLG	Eg12	Present study
ADSSSASGQQQESGVPSQSDQASTSSQLG	Eg13	Present study
ADSSSASGQQQESGVSSQSDASTSSQLG	Eg14	Present study
ADSSSASGQQQESSVPSQSGASTSSQLG	Eg15	Present study
ADSSSASGQQQESGVPSQSGASTSSQLG	Eg16	Present study
AGSSSAGGQQQESSVSSQSDQASTSSQLG	Eg17	Present study
ADSSSAGGQQQESSVSSQSGQASTSSQLG	Eg18	Present study
ADSSSASGQQQESSVLSPSGQASTSSQSG	Eg19	Present study
ADSSSASGQQQESGVLSQSGQASTSSQSGT	Eg20	Present study
AGSSSASGQQQESGVSSQSEQASTSSQLG	Eg21	Present study
TDSSSASGQQQESGVPSQSGQASTSSQLG	Eg22	Present study
ADSSSAGGQQQESSVSSQSDQASTSSQLG	Eg23	Present study
ADSSSASGQQQESSVSSQSGQASTSSQLG	Eg24	Present study
TDSSSTSGQQQESSVLSQSDQASTSSQSG	Eg25	Present study

## Discussion

*Anaplasma marginale* is one of the most important tick-borne pathogens worldwide [39]. All animals investigated in this study were infested with ticks without showing clinical signs of anaplasmosis. The ticks were identified as *Hyalomma excavatum* and *Rhipicephalus annulatus*, both species being incriminated as vectors for *A. marginale* [40, 41].

The examination of Giemsa-stained blood smear was less sensitive compared to PCR-based detection methods potentially due to sampling subclinical or persistently infected animals that often show low numbers of infected erythrocytes. Another cause could be the dependency of the method for the microscopic visualization of *A. marginale* intraerythrocytic stage [42–45]. ELISA registered high sensitivity and low specificity compared to qPCR, being based on the detection of antibodies that occur during the persistent infection. Moreover, it depends on animal’s health and the ability of its immune system to produce antibodies against this variable antigenic pathogen.

Reverse line blot technique was highly sensitive and specific. Real time PCR was the most sensitive assay for detecting *A. marginale.* Previous studies registered similar findings, recommending molecular techniques for diagnosis of anaplasmosis [24]. These methods can overcome the persistent nature of the infection and the antigenic variability of the pathogens which adversely affect the ability of the serological tests to detect the infection [9, 24, 39].

Buffaloes in Middle and Upper Egypt showed lower infection rates compared to cattle from the same regions. These results could indicate a natural resistance against *A. marginale* in Egyptian buffaloes. Previous studies also stated that water buffaloes have the ability to reduce the infection and multiplication of this pathogen in the cells and also have natural resistance against tick infestation, reducing the probability of transmission of tick-borne pathogens [46–48]. Without clearing the persistent infection, buffaloes’ immune system can offer protection against the high level of rickettsemia and the acute phase of the disease if the animal is challenged with the homologous strain [49].

The prevalence of *A. marginale* was analyzed based on several risk factors related to host (age, sex, species and breed), environment, seasonal variation and study area. The sex of the animals did not affect the susceptibility. A study from Pakistan also did not find significant difference in the animals’ susceptibility according to their sex, but mainly linked with the degree of tick infestation. Animals exposed to heavy infestations were usually at higher risk than those exposed to light tick infestations [50]. Animal age was also among the factors affecting the probability of this infection in different animals. Older animals were more often infected than young ones. A previous study in Southern Queensland, also concluded that animals older than one year are more susceptible to the infection than younger animals [51]. On the contrary, a study in Pakistan concluded that animal susceptibility is not influenced by the age. Another study in Brazil concluded that animals below six months are more susceptible compared with older ones [12, 52]. Similar results to our findings regarding the cattle breeds were reported in Pakistan, confirming the lack of significance between infection rates of different cattle breeds [50]. Prevalence of *A. marginale* in Egypt was not affected by seasonal variation as suggested also in one study from Brazil [45] but in disagreement with a study in Southern Queensland which revealed that the disease is usually common during non-hot months (autumn and winter) [46]. The different localities did not affect the prevalence of *A. marginale* as in a previous study in Pakistan [50]. Based on these epidemiological findings and the genetic variability of *A. marginale* detected in different localities from previous studies [28, 39], we can conclude that prevalence and epidemiological features of *A. marginale* infection is closely related to its geographical distribution, each region having different genotypes.

Co-infections with *A. marginale* and pathogens such as *T. annulata*, *B. bovis*, *B. bigemina*, *B. occultans* and *A. platys* were found in both cattle and buffaloes. *Anaplasma platys* and *B. occultans* were detected for the first time in Egypt. Although *A. platys* is described as a tick-borne pathogen of dogs [53], it was previously also detected in cattle from China [54]. The co-infection with *A. marginale* and pathogens like *Theileria* spp., *Babesia* spp. and other *Anaplasma* species is a common finding and could be attributed to mixed infestation with several tick species or the ability of individual species to carry multiple pathogens [4, 13–15].

The phylogenetic analysis of *A. marginale* based on *msp1α* gene revealed that the Egyptian isolates were not only different from those circulating in other countries but also different from each other. The genetic differences found in the Egyptian isolates were independent of their locality. The occurrence of similar isolates in different localities may be a result of the uncontrolled movement of live animals between different localities in Egypt for marketing and slaughter. Although there is a marked difference between the Egyptian strains and other strains circulating worldwide, some strains are similar to isolates circulating in South Africa and Brazil perhaps due to importation of live animals from different countries to Egypt. Official data report for 2018 of the United States Department of Agriculture (USDA) stipulated that the importation of live animals is steadily increasing, and the same report revered that Sudan and Brazil are the largest suppliers for the live cattle.

The analysis of *msp1α* microsatellite sequences confirmed the presence of different genotypes amongst *A. marginale* strains obtained from both cattle and buffaloes from different localities in Egypt. Analysis of the translated sequences revealed that there were 25 new tandem repeats in the Egyptian isolates (Eg1 to Eg25), resulting in 15 different *A. marginale* genotypes. The two tandem repeats (F) and (τ) were previously isolated from USA, Israel, Cuba and Brazil, countries that act as suppliers of imported living animals to Egypt [55]. The Brazilian TR (τ) was found in cattle from Egyptian oases and the TR (F) from USA was found in cattle from Middle Egypt where there are some farms for the imported animals. These TRs found in different forms, include the original copy and another form with slight mutations that confirmed the occurrence of mutations to help the pathogens to adapt in the Egyptian field. In addition, the presence of predominant genotype, with the following TRs (Eg17, Eg23, Eg23, Eg23 and Eg24) in cattle from Upper Egypt (Assiut Governorate) and Egyptian oases (New Valley Governorate), is another evidence for the adverse effects of the uncontrolled animal movements which should be avoided by the veterinary authorities in Egypt. Water buffaloes are important hosts for *A. marginale* and can act as reservoir and transmit the infection to ticks. No genetic similarity was found in the *msp1α* gene structure of *A. marginale* isolated from cattle and water buffaloes except for one TR (Eg3) isolated from both species from Egyptian oases (MN370077, NV2). This finding could indicate that buffaloes act as reservoirs for specific genotypes which are not directly transmitted to cattle except after subjected to mutations. These mutations could make them infective and pathogenic for cattle especially in endemic areas and in mixed farms. The same hypothesis was indicated in previous studies stating that cattle may acquire *A. marginale* in superinfection with more than one genotype leading to generation of new genotypes. This process is sustained by the fact that antigenically, *A. marginale* is a highly variable pathogen, and these studies confirmed the use of this mechanism by *A. marginale* to escape the host immunity for persistence [56, 57].

## Conclusions

In conclusion, infections with *Anaplasma marginale* were common in all the investigated areas from Egypt. Buffaloes were less often positive than cattle. Age of the animal was the main risk factor for positivity. Most isolates of *A. marginale* in Egypt were genetically diverse according to locality and host species. *Anaplasma platys* and *B. occultans* were detected in both cattle and buffaloes for the first time in Egypt. The qPCR and RLB assays are sensitive and specific for detection of *A. marginale* infection. Fifteen new Egyptian genotypes of *A. marginale* were identified in both cattle and buffaloes in Egypt.

## Supplementary information

**Additional file 1: Figure S1.** Phylogenetic tree inferred by using the Neighbor-Joining method, evolutionary distances were computed using the Kimura 2-parameter model in MEGA X and *A. phagocytophilum* (HG528610) as the outgroup.

## Data Availability

All data analyzed and generated during this study are included in this published article. The newly generated sequences were submitted to the GenBank database under the following accession numbers: *18S* rRNA gene (*T. annulata*: MN223723-MN223737; *B. bigemina*: MN227676-MN227679; *B. occultans*: MN227675); *GroEL*/*16S* rRNA genes (*A. platys*: MN202017-MN202023 and MN227688); and MSP1 α gene (*A. marginale*: MN227687, MN227689-MN227692).

## Abbreviations

ELISA: enzyme-linked immunosorbent assay
PCR: polymerase chain reaction
qPCR: real time PCR
RLB: reverse line blot
msp1α: major surface protein α1
TRs: tandem repetitions
Hi-Di: highly deionized formamide
NCBI: National Center for Biotechnology Information
BLAST: basic Local Alignment Search Tool
SD: putative Shine-Dalgarno
Eg: Egyptian genotype
TP: true positive
TN: true negative
FP: false positive
FN: false negative
PPV: positive predictive value
NPV: negative predictive value
CPV: combined predictive value
USDA: United States Department of Agriculture
