# Supplementary figures and images for: Epidemiology and genotyping of Anaplasma marginale and co-infection with piroplasms and other Anaplasmataceae in cattle and buffaloes from Egypt
Source: Parasit Vectors. 2020 Sep 29;13:495. doi: 10.1186/s13071-020-04372-z (PMC7526245; doi:10.1186/s13071-020-04372-z)

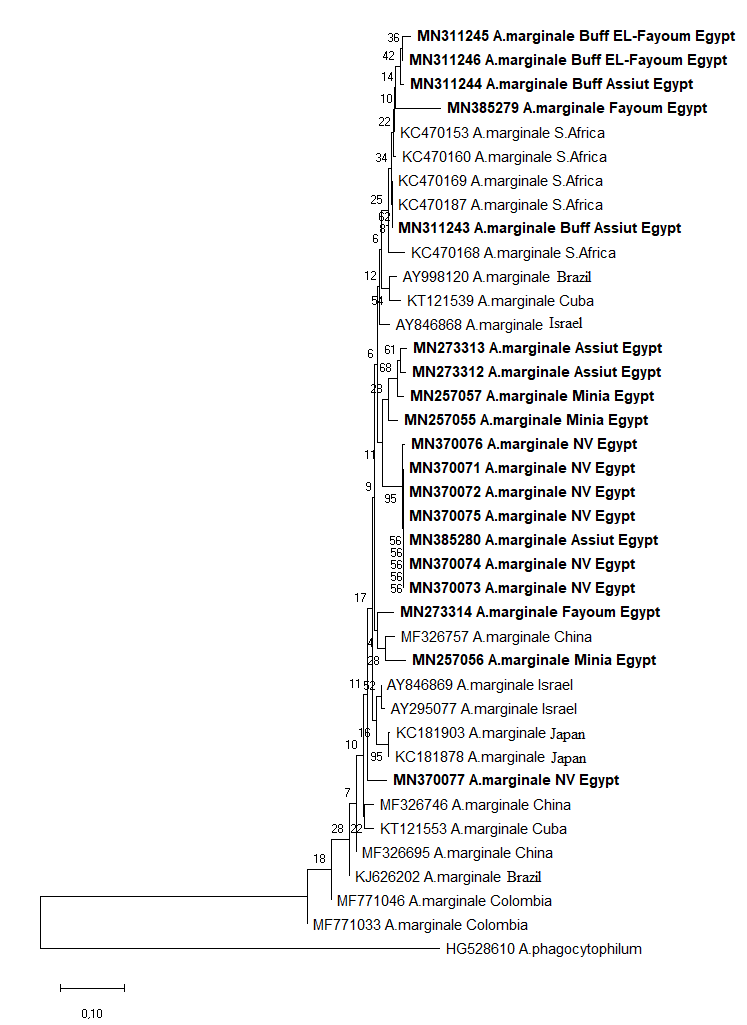

Supplement: Supplementary file 1 — Additional file 1: Figure S1. Phylogenetic tree inferred by using the Neighbor-Joining method, evolutionary distances were computed using the Kimura 2-parameter model in MEGA X and A. phagocytophilum (HG528610) as the outgroup. [file 13071_2020_4372_MOESM1_ESM.tif]
